# Influenza and Pregnancy: A Review of the Literature from India

**DOI:** 10.1155/2015/867587

**Published:** 2015-02-24

**Authors:** Ashwini Bhalerao-Gandhi, Pankdeep Chhabra, Saurabh Arya, James Mark Simmerman

**Affiliations:** ^1^P. D. Hinduja Hospital and Medical Research Centre, Veer Savarkar Marg, Mahim, Mumbai 400016, India; ^2^Sanofi Pasteur India Pvt. Ltd., 54/A, Mathuradas Vasanji Road, Andheri East, Mumbai 400093, India; ^3^Sanofi Pasteur Thailand, 87/2 CRC Tower, 23rd Floor, Wireless Road, Bangkok 10330, Thailand

## Abstract

Maternal influenza infection is known to cause substantial morbidity and mortality among pregnant women and young children. Many professional healthcare bodies including the World Health Organization (WHO) have identified pregnant women as a priority risk group for receipt of inactivated seasonal influenza vaccination. However influenza prevention in this group is not yet a public health priority in India. This literature review was undertaken to examine the Indian studies of influenza among pregnant women. Eight Indian studies describing influenza burden and/or outcomes among pregnant women with influenza were identified. In most studies, influenza A (pH1N1) was associated with increased maternal mortality (25–75%), greater disease severity, and adverse fetal outcomes as compared to nonpregnant women. Surveillance for seasonal influenza infections along with higher quality prospective studies among pregnant women is needed to quantify disease burden, improve awareness among antenatal care providers, and formulate antenatal influenza vaccine policies.

## 1. Introduction

Influenza is an acute, viral respiratory infection that causes significant morbidity and mortality among high-risk groups like pregnant women and infants. Physiological and immunological changes during pregnancy including decreased tidal volume and lung capacity, increased oxygen consumption and cardiac output, and selective suppression of T-helper-type 1 cell-mediated immunity that impairs maternal response to infection place pregnant women at high risk of complications and hospitalizations [1–3]. During the influenza pandemics of 1918, 1957, and 2009, pregnant women were at high risk of complications such as primary viral influenza pneumonia and mortality [4–6]. Even during the interpandemic period, pregnant women have been observed to be at a 18-fold higher risk of hospitalization as compared to healthy nonpregnant women and the risk is greatest among women in later stages of pregnancy [7–9]. Pregnant women with coexisting medical conditions such as asthma or diabetes are at 3-4 times greater risk of morbidity as compared to nonpregnant control subjects with similar high-risk conditions [7, 10].

Influenza infection among pregnancy is also linked to adverse pregnancy and birth outcomes. Newsome and colleagues reported that among severely ill pregnant women hospitalized for pandemic influenza A (pH1N1), 63.6% delivered preterm and 43.8% delivered low birth weight infants, compared with US averages of 12.3% for preterm birth and 8.2% for low birth weight [11]. Pregnant women hospitalized for respiratory illness during influenza season had higher odds of preterm delivery (adjusted OR 3.82, 95% CI 3.53–4.14), cesarean delivery (adjusted OR 3.47, 95% CI 3.22–3.74), and fetal distress (adjusted OR 2.33, 95% CI 2.15–2.52) and their infants were more likely to be small-for-gestational age (adjusted relative risk of 1.66; 95% CI 1.11–2.49) and have lower mean birth weight (3448.5 ± 498.2 versus 3531.3 ± 504.1 g; *P* < 0.009) as compared to pregnant women not hospitalized for respiratory illness [12, 13].

Among young infants, influenza infection may present with fever and other nonspecific symptoms such as irritability, reduced oral intake, dyspnea, vomiting, and diarrhea. In a prospective study in Bangladesh where influenza virus circulates throughout the year, about one-third of infants <6 months had serological confirmation of influenza infection [14]. In an urban setting in Dhaka, Bangladesh, 28% of influenza-positive children had pneumonia, whereas influenza virus was associated with 10% of all childhood pneumonia [15]. In a community-based study in Bangladesh among infants <6 months old, the incidence of acute respiratory infection and pneumonia due to influenza virus was 6/100 child-years (95% CI: 3–12) and 3/100 child-years (95% CI: 1–8), respectively [16]. The interaction between influenza virus and pneumococcus can increase the burden and severity of pneumonia in children, especially in the tropical countries where pneumonia is highly prevalent [17, 18].

Trivalent influenza vaccines (TIV) contain antigens from two influenza A strains (H1N1 and H3N2), and one of the two influenza B strains (Yamagata or Victoria), as recommended by WHO [19]. Immunologic responses generated by influenza immunization in pregnant women are comparable to nonpregnant women and can provide protection to the fetus and infant by efficiently transferring influenza-specific antibodies across the placenta [20–23]. In the Mother Gift's randomized blinded trial conducted in Bangladesh among 340 pregnant women in the third trimester, maternal influenza vaccination led to a 63% reduction of laboratory-confirmed influenza, 29% reduction in febrile respiratory illness among infants, and a 36% reduction in febrile respiratory illness among mothers [24]. During the period of influenza virus circulation, maternal influenza vaccination was associated with statistically significant reductions in febrile respiratory illness among mothers and infants (*P* = 0.0003), higher mean birth weight in infants (*P* = 0.02), and lower proportion of infants who were small-for-gestation age (*P* = 0.03) [25]. No differences were observed between the intervention (influenza vaccine) and control (pneumococcal polysaccharide vaccine) groups for adverse fetal, perinatal, or infant outcomes, and none of the adverse events reported were found to be related to the influenza vaccine [24]. Published reviews of observational studies and clinical trials from the past few decades provide reassuring results on the safety of seasonal influenza vaccines among pregnant women and infants [26–28].

The WHO Strategic Advisory Group of Experts (SAGE) on immunization has given highest priority to pregnant women for receiving inactivated influenza immunization [29]. In India, influenza vaccine uptake among pregnant women is extremely poor (12.8% for pandemic influenza vaccine and none for seasonal influenza) [30, 31]. The lack of disease burden data among pregnant Indian women may have contributed to the perception that prevention of influenza in pregnant women and young children may not be a public health priority. The objective of this review is to examine the published Indian studies to better understand the burden of influenza among pregnant women.

## 2. Review of Indian Studies

### 2.1. Methods

A literature search was conducted in PubMed, EMBASE, SCOPUS, and Medind databases and the website of Indian journals (http://www.indianjournals.com) to identify Indian studies that were published till 31 December 2013 and described burden of influenza and/or outcomes among pregnant women with seasonal or pandemic influenza. The search terms used included “influenza,” “India,” and “pregnancy.” Wherever appropriate, the search also included the term “pregnan^*^” to capture any forms of the word “pregnant” or “pregnancy.” After eliminating duplicates, titles and abstracts of citations were screened for relevance and were excluded if they did not focus on influenza epidemiology in India. On excluding review articles, editorials, case reports, and letters, potentially relevant citations included research studies that described influenza infection among pregnant women. Full texts of the potentially relevant citations and of the citations in which information was unclear in the abstract were retrieved. Finally, references of all selected articles were examined to identify additional articles.

Studies were included in the final review if they provided the number of pregnant women with influenza infection along with the data on comorbidities, maternal/fetal outcomes, trimester distribution, and severity of infection.

### 2.2. Results

#### 2.2.1. Study Characteristics

Of 272 citations identified in our search, eight studies were included in the final review [32–39]. All studies were initiated during the pandemic of 2009-2010 in various regions of India (five studies were conducted in southern India, two studies in western India, and one study in northern India). Pregnant women were described as part of the overall population tested for influenza. In all studies, suspected influenza like illness (ILI)/severe acute respiratory illness (SARI) cases were confirmed for influenza by reverse transcriptase polymerase chain reaction (RT-PCR) test. All studies primarily focused on diagnosis of pandemic influenza A (pH1N1) and only two studies described prevalence of other seasonal influenza viruses [32, 33]. Three studies described the prevalence of influenza among both outpatients and inpatients [34, 35, 37], while five others focused only on severely ill patients requiring hospitalization [32, 33, 38, 39] or intensive care [36]. Only two studies provided information on influenza positivity among nonpregnant females [33, 34]. Gunasekaran observed important differences between seasonal and pandemic influenza positivity among pregnant and nonpregnant women [33]. Overall, a higher proportion of pregnant women were positive for both seasonal influenza (11.1% pregnant versus 1.4% nonpregnant) and influenza A (pH1N1) (21.4% pregnant women versus 2.7% nonpregnant). Pramanick et al. reported that, among all women presenting with ILI/SARI, influenza A (pH1N1) was positive in 25.3% of pregnant/puerperal women and 29.6% of nonpregnant women [34]. Most pregnant women with influenza A (pH1N1) infection presented to the hospital during second or third trimester. The study characteristics are described briefly in Table 1.

#### 2.2.2. Outcomes

*Maternal Mortality.* Overall, the studies included in this review showed that pandemic influenza was associated with high mortality among Indian pregnant women. Across five studies, influenza-related maternal mortality ranged from 25% to 70% among pregnant women [34–38], whereas Gunasekaran reported mortality of 3.7% [33]. In the descriptive study by Sharma and colleagues, 2 out of eight women who died due to influenza A (pH1N1) were pregnant [39]. Pramanick and colleagues reported that mortality among pregnant women with influenza was associated with a significant delay in presentation to hospital (median time: 6 days for those who died versus 1.5 days for survivors), dyspnea, need for ICU admission, need for mechanical ventilation, and renal failure [34]. Mathur et al. observed a higher maternal mortality rate among women in third trimester (80%) as compared to women presenting in early pregnancy (63%) [37]. Mehta et al. reported a 2.9-fold higher mortality among pregnant women that was not statistically significant (OR = 2.90 (95% CI: 0.48–17.72)) [38]. Similarly, Ramakrishna et al. did not find a statistically significant difference in mortality among pregnant and nonpregnant women [36]. However, high mortality due to influenza A (pH1N1) among pregnant women was observed by Puvanalingam (25% pregnant women versus 2.7% nonpregnant), Pramanick (25% pregnant women versus 8.3% nonpregnant women, *P* = 0.04), and Mathur (70% pregnant women versus 26% nonpregnant).

*Fetal and Perinatal Outcomes.* Three studies reported the effect of maternal influenza infection on fetal and perinatal outcomes. These studies reported fetal mortality ranging from 5.5% to 33% and prematurity rates were from 20% to 33% [34, 35, 38].

*Severity of Disease, Comorbidities, and Complications.* Four studies provided data on severity, comorbidities, and complications due to influenza infection among pregnant women [32–35]. Out of 20 pregnant and postpartum women with influenza A (pH1N1) infection, Pramanick reported that 8 (40%) required ICU admission, 4 (20%) developed renal failure, and 1 (5%) developed seizures. Chudasama and colleagues reported ICU admission among 73% of pregnant and postpartum women. Palani et al. reported that 11% of pregnant women with influenza A (pH1N1) were admitted to the ICU for the management of severe outcomes. A higher proportion of pregnant women with seasonal influenza (21.4%) had asthma in comparison to pregnant women with influenza A (pH1N1) (3.7%). Puvanalingam observed that 75% of pregnant women with influenza had pneumonia in comparison to 24% nonpregnant women. Additional risk factors such as diabetes mellitus and obesity which are known to complicate pregnancy along with influenza A (pH1N1) were not described specifically among pregnant women in all studies.

## 3. Discussion

The eight studies that assessed the effect of influenza among pregnant Indian women were conducted in response to the 2009 influenza pandemic and only one assessed seasonal influenza infections in the postpandemic phase.

The maternal mortality rate observed in most Indian studies was higher than that reported in other countries [6, 40–42]. The mortality estimates in the Indian studies varied widely (3.7% to 70%) which could be due to the diverse study populations and differing sampling methods used.

Delay in the initiation of antiviral treatment and the presence of underlying comorbidities are associated with severe H1N1 disease [43], but not all studies provided information on these factors. Receipt of Oseltamivir to influenza-positive patients was reported in six studies, but whether it was given within the recommended duration from onset of symptoms was not mentioned in all studies. Similarly, information on comorbidities specifically among pregnant women subgroup was mentioned in only two studies [34, 35]. Other factors prevalent in developing countries that may contribute to severe disease are lack of awareness and misconceptions regarding influenza, poor diagnostic and intensive care management, and use of over-the-counter medications [44].

The finding that pregnant women with influenza (pH1N1) infection present with severe disease later in pregnancy is consistent with other studies. In a Singaporean study, women presenting in the second trimester had a 1.2-fold increase and women in the third trimester had 2.3-fold higher odds of hospitalization [45]. A similar finding was observed in a review by Liu et al. in which the results from seven studies from different geographic areas revealed that 9.1% of the cases with influenza A (pH1N1) infection occurred in the first trimester, 29.8% in the second trimester, and 47.0% in the third trimester [46].

This literature review has some limitations. We excluded studies that provided only the number of pregnant women without any other information for this group and studies that were presented only in conferences or meetings as abstracts. Among two studies that attempted to describe prevalence of seasonal influenza among pregnant women, one overestimated the burden of seasonal influenza by misclassifying patients that were negative for pandemic influenza A (pH1N1) as patients with seasonal influenza without actually testing them for influenza A (H3N2) or influenza B [32]. The other study did not describe characteristics or outcomes among women positive for influenza A/H3N2 or influenza B viruses [33].

There were methodological limitations in the included studies. Pregnant women were described as a subgroup of a population with ILI/SARI that was referred to tertiary-level hospitals, thus capturing more severe cases, while milder influenza infections might not have been captured. Some of these studies lacked power to assess the association between influenza and adverse maternal and/or fetal outcomes. Studies were also limited by retrospective study designs, short follow-up periods, small sample sizes, inadequate assessment of maternal and neonatal outcomes, and inadequate description of comorbidities or treatment. Some studies described combined outcomes among pregnant and postpartum women preventing comparisons between these two groups.

## 4. Conclusions

Though limited data are available from India, this review highlighted the high burden of maternal and fetal complications during pregnancy, especially due to pandemic Influenza A (pH1N1). However, six years after the pandemic, influenza A (pH1N1) strain continues to circulate as a seasonal virus and continues to cause outbreaks, severe illness, and some deaths [47, 48]. Since most studies did not test for seasonal influenza virus (A/H3N2 or type B virus) among pregnant women, this review identified an important data gap that can be addressed by surveillance of all influenza virus serotypes circulating in India. Additionally, better quality prospective studies to assess influenza burden among pregnant women are needed to help improve awareness among antenatal care providers and better inform antenatal influenza vaccine policy in India.

## Figures and Tables

**Table 1:** Characteristics of included studies.

Sr. number	Author (year)	Objective	Region	Setting	Sample	Period	Outcomes	Results
1	Mathur et al. (2013) [37]	To assess the clinical profile, factors determining the response, prognosis of the disease, and outcome in H1N1 positive patients during 2009-2010 H1N1 pandemic	Jodhpur (West India)	Hospital-based, ICU/isolation patients	Overall population: 221 Pregnant women: 37	2009-2010	Clinical and epidemiological characteristics	**Prevalence of Influenza virus:** (i) Influenza A (pH1N1): 37 (23.4%) **Length of pregnancy at presentation:** (i) First trimester: 4 (11%) (ii) Second trimester: 18 (49%) (iii) Third trimester: 15 (40%) **Maternal mortality:** (i) 26 (70%) pregnant women died (ii) Mortality in third trimester: 80% **Treatment:** (i) All patients received Oseltamivir, Zanamivir, oxygen and ventilatory therapy, intravenous antibiotics and fluids, low dose steroids, and symptomatic therapy according to WHO guidelines

2	Mehta et al. (2013) [38]	To observe clinical profile of admitted patients with confirmed H1N1 swine flu infection and risk factors associated with the need of mechanical ventilation and/or death	Kochi, Kerala (South India)	Hospital-based inpatient	Overall population: 115 Pregnant women: 6	August 2009 to December 2011 (28 months)	Clinical and epidemiological characteristic Risk factors associated with mechanical ventilation/death	**Influenza virus prevalence:** (i) Influenza A (H1N1): 5.2% **Maternal mortality**: 2 women (33%) **Loss of fetus**: 2 women (33%) **Preterm delivery**: 2 women (33%) **Treatment:** All patients received Oseltamivir 75 mg twice daily for 5–10 days as per CDC guidelines

3	Ramakrishna et al. (2012) [36]	To detail the profile and outcomes of patients admitted to the intensive care unit (ICU) with pandemic Influenza A (H1N1) 2009 virus [P(H1N1)2009v] infection	Bangalore, Vellore, Manipal (South India)	Hospital-based, ICU patients	Overall population: 1902 Pregnant women: 19	September 2009–December 2009 (4 months)	(i) Hospital mortality (ii) Need for any duration of ventilation, tracheostomy, and skeletal muscle relaxants (iii) Need for renal replacement therapy (iv) Duration of ICU (v) Hospital stay	**Onset of infection:** (i) Antepartum: 14 women (ii) Postpartum: 5 women **Maternal mortality:** (i) 10 pregnant/postpartum women (52.6%) died (ii) Other outcomes were not described separately for pregnant women **Treatment:** All patients received Oseltamivir; no separate mention of timing of initiation of Oseltamivir in pregnant women

4	Gunasekaran (2012) [33]	To determine the cumulative prevalence of H1N1 2009 influenza among pregnant and postpartum women in Tamil Nadu	Chennai (South India)	Hospital-based, inpatient and outpatient	Overall population: 7638 Pregnant women: 126	September 2009–May 2011 (21 months)	Clinical and epidemiological characteristics	**Prevalence of influenza virus:** (i) Influenza (pH1N1): 27 women (21.4%) (ii) Influenza A/H3: 12 women (9.5%) (iii) Influenza B: 2 women (1.6%) **Onset of infection:** (i) Antepartum: 12 women (ii) Postpartum: 15 women **ICU admission and complication:** (i) Admitted in ICU and treated for severe outcomes: 3 (11.1%) (ii) Admitted in obstetric ward and treated for moderate outcomes: 5 (18.5%) women **Maternal mortality:** (i) One woman died during postpartum period **Treatment:** Oseltamivir was initiated in patients considering their high-risk status

5	Pramanick et al. (2011) [34]	To assess the clinical profile of pregnant/puerperal women infected with pandemic H1N1 influenza A and to evaluate their outcome	Vellore (South India)	Hospital-based	Overall population: 566 Pregnant women: 20	August 2009–January 2010 (6 months)	Primary outcome (i) Maternal mortality Secondary outcomes (ii) Need for ICU admission (iii) Need for mechanical ventilation (iv) Renal failure	(i) Out of 79 pregnant women, 20 (29%) women presenting with ILI/SARI were pregnant **Onset of infection:** (i) Antepartum: 16 women (80%) (ii) Postpartum: 4 women (20%) **Length of pregnancy at presentation:** (i) Second trimester: 4 women (20%) (ii) Third trimester: 11 women (55%) (iii) 1 woman had molar pregnancy at 18 weeks of pregnancy **ICU admission:** (i) Required by 8 pregnant/postpartum (40%) **Maternal mortality**: 5 women (25%) died **Fetal mortality**: 1 **Complications:** (i) Renal failure: 4 women (20%) (ii) Mechanical ventilation: 7 women (35%) (iii) Seizures: 1 woman (5%) **Treatment:** Oseltamivir received by 18 women (90%); no mention of timing of initiation of Oseltamivir

6	Puvanalingam et al. (2011) [35]	To study clinical profile of H1N1 influenza cases and to study the impact of H1N1 infection on pregnancy outcome	Chennai (South India)	Hospital-based inpatient and outpatient	Overall population: 442 Pregnant women: 12	August 2009–January 2010 (6 months)	Clinical characteristics and outcome of pregnant women	**Length of pregnancy at presentation:** (i) First trimester: 2 (16.7%) women (ii) Second trimester: none (iii) Third trimester: 10 (83.3%) women **Maternal mortality:** 3 women (25%) died **Complication:** (i) 9 women (75%) presented with pneumonia **Fetal death:** (i) 2 women (16.7%) had spontaneous abortion following intrauterine death in the third trimester Treatment: not mentioned

7	Chudasama et al. (2010) [32]	To investigated the clinicoepidemiological characteristics of patients who were hospitalized with 2009 pandemic H1N1 influenza virus infection and seasonal influenza in the Saurashtra region of India	Rajkot (West India)	Hospital-based inpatient	Overall population: 773 Pregnant women: 15	September 2009–February 2010 (6 months)	Clinical and epidemiological characteristics	**Length of pregnancy at presentation:** (i) All women presented in either 2nd or 3rd trimester **ICU admission:** (i) 11 women (73.3%) required intensive care **Treatment:** All patients received Oseltamivir, no separate mention of number of pregnant women that received the drug within 2 days of illness onset

8	Sharma et al. (2010) [39]	To describe the mortality data of severe influenza cases admitted in a tertiary care center in New Delhi	New Delhi (North India)	Hospital-based inpatient	Total: 8 women Pregnant women: 2	September 2009 to January 2010 (5 months)	Mortality	Out of 8 deaths due to influenza A (pH1N1), 2 women were pregnant **Length of pregnancy at presentation:** (i) First trimester: one woman (ii) Postpartum period: one woman, child delivered by caesarean section
